# Non-serotinous woody plants behave as aerial seed bank species when a late-summer wildfire coincides with a mast year

**DOI:** 10.1002/ece3.1247

**Published:** 2014-09-17

**Authors:** Edith Pounden, David F Greene, Sean T Michaletz

**Affiliations:** 1 Department of Biology, Concordia UniversityMontréal, Québec, H3G 1M8, Canada; 2 Department of Geography, Planning and Environment, Concordia UniversityMontréal, Québec, H3G 1M8, Canada; 3 Department of Ecology and Evolutionary Biology, University of ArizonaTucson, Arizona, 85721

**Keywords:** Adaptation, Engelmann spruce, fire, mass seeding, masting, regeneration, seed viability, serotiny

## Abstract

**Abstract:**

1. Trees which lack obvious fire-adaptive traits such as serotinous seed-bearing structures or vegetative resprouting are assumed to be at a dramatic disadvantage in recolonization via sexual recruitment after fire, because seed dispersal is invariably quite constrained. We propose an alternative strategy in masting tree species with woody cones or cone-like structures: that the large clusters of woody tissue in a mast year will sufficiently impede heat transfer that a small fraction of seeds can survive the flaming front passage; in a mast year, this small fraction would be a very large absolute number.
2. In Kootenay National Park in British Columbia, we examined regeneration by Engelmann spruce (*Picea engelmannii*), a non-serotinous conifer, after two fires, both of which coincided with mast years. Coupling models of seed survivorship within cones and seed maturation schedule to a spatially realistic recruitment model, we show that (1) the spatial pattern of seedlings on a 630 m transect from the forest edge into the burn was best explained if there was in situ seed dissemination by burnt trees; (2) in areas several hundred meters from any living trees, recruitment density was well correlated with local prefire cone density; and (3) spruce was responding exactly like its serotinous codominant, lodgepole pine (*Pinus contorta*).
3. We conclude that non-serotinous species can indeed behave like aerial seed bank species in mast years if the fire takes place late in the seed maturation period. Using the example of the circumpolar boreal forest, while the joint probability of a mast year and a late-season fire will make this type of event rare (we estimate *P* = 0.1), nonetheless, it would permit a species lacking obvious fire-adapted traits to occasionally establish a widespread and abundant cohort on a large part of the landscape.

## Introduction

Woody plants in many parts of the world cope with a disturbance regime dominated by large, stand-replacing fires (e.g., Johnson 1992; Veblen et al. 1994; Peet 1999). Indeed, these fires are so large that, given dispersal constraints, species which must recolonize from the edge are at a tremendous disadvantage in the early recruitment phase (Greene and Johnson 2000). There are three fire-adaptive traits that permit in situ establishment and thus allow a species to circumvent this dispersal constraint (Keeley et al. 2011). The first is asexual reproduction. This is particularly advantageous, but much less common, when the perennating buds are located on widespread roots (e.g., in aspen, *Populus tremuloides*). More typically, angiosperms reliably sprout from the root collar (few gymnosperm species can do this), a trait often enhanced in fire-adapted species by the presence of lignotubers, storage organs at the root–shoot junction which contain numerous dormant buds (James 1984). The second trait is an aerial seed bank. Persistent ovulate cones or inflorescences ensure that a large proportion of seeds are on hand to disperse from burnt trees or shrubs onto the receptive seedbeds created by combustion of organic soil layers (Greene et al. 1999). Aerial seed banks are common among plants in many fire-prone areas, including several *Pinus* species in the Northern Hemisphere and *Banksia* species in Australia (Lamont et al. 1991). The third trait is a soil seed bank. Soil seed banks that can persist through fire, while common in Mediterranean and desert ecosystems, are, however, quite rare among species in the circumboreal forest where smoldering combustion of the deep organic layer typically kills all seeds (Johnson and Fryer 1996).

How do species lacking these three traits persist in fire-prone environments? They must seed in from the fire edge or from residual stands within the burn; empirically, we find very few seedlings at distances greater than about 100 m from surviving seed sources (Zasada 1985; Greene and Johnson 1996). It remains a mystery how species without a strong asexual response or either aerial or soil seed banks can persist in a landscape dominated by stand-replacing fires (Greene and Johnson 2000). We hypothesize that under certain conditions, a species can respond to stand-replacing fire as if it possessed an aerial seed bank, dispersing viable seeds from dead or dying trees within the interior of the burn. Four conditions must be met for this hypothesis to be correct. First, seeds must be contained within structures that provide resistance to heat transfer from incident heat flux and can sufficiently insulate the seeds against lethal temperatures. Structures capable of insulating seeds from fire vary by species according to their thermophysical properties and can in some cases be single ovulate cones or fruits (Mercer et al. 1994; Michaletz et al. 2013) or, in other cases, dense clusters of many cones or fruits (Judd and Ashton 1991). Second, fire must occur when seeds are either mature or sufficiently advanced that they can finish maturation even when the tree is dead. Third, the fire must not occur so late in the year that the cones or fruits have begun to open; otherwise, the cones or fruits and any enclosed seeds will combust as fine fuels. For example, Michaletz et al. (2013) concluded that the window between sufficient maturation and cone opening in the North American boreal species white spruce (*Picea glauca*) was, empirically, about 600 to 1180 degree days.

The fourth requirement is that the species must be enjoying a mast year at the time of the fire (Koenig and Knops 1998; Greene and Johnson 2004). Masting is a condition in which individual plants of a species synchronously produce unusually high numbers of fruit or seeds over a wide region (Silvertown 1980; Kelly 1994). High production in one season (a mast year) is typically followed by several years of low seed production. We consider a mast year an additional requirement because high seed mortality will certainly occur during passage of the flaming front, and there will be therefore little in situ recruitment in a non-mast year. The coincidence of a mast year and a fire will have an augmenting effect from the living plants at the edge: The good seedbeds created by smoldering combustion are ephemeral, lasting only a few years before leaf accrual renders them poor substrates for small-seeded species (Greene and Johnson 2000; Charron and Greene 2002; Peters et al. 2005), and thus, the edge trees would have a much stronger input (albeit still distance limited) in a mast year.

Our objective is to formally model and test the hypothesis that a species can perform as if it had an aerial seed bank if a fire takes place late in the seed maturation period during a mast year. We focus on the conifer, Engelmann spruce (*Picea engelmannii* Parry ex Engelm.), in the southern Canadian Rockies following two late-summer fires. This is a masting species, with very little seed produced in inter-mast years (Fowler and Roche 1976; Greene and Johnson 2004). Both fires were selected for this study because they coincided with mast years. As Engelmann spruce is extremely closely related to, and hybridizes extensively with, white spruce (Daubenmire 1974; Bouillé et al. 2011), we can posit the same degree day window for seed maturation mentioned above. Likewise, reproductive potential and interannual variation in crop sizes are comparable between Engelmann and white spruce, with both species predictably showing a decrease in seed production with elevation (Owens and Molder 1984; Greene and Johnson 2004). Unpublished data collected by Greene and Pounden in consecutive years (2005 to 2013) show that the cone crops of the two species are indeed highly positively correlated.

We will also compare recruitment of Engelmann spruce with the aerial seed bank species, lodgepole pine (*Pinus contorta* Dougl. ex Loud. var. *latifolia* Engelm.). We develop a general mechanistic model of sexual recruitment applicable to any species with or without aerial seed banks, or with a combination of the two responses. Our model makes two predictions for these late-summer fires. First, unlike the steep negative exponential decline in seeds or seedlings of wind-dispersed species from burn edges due to the dispersal constraint (Greene and Johnson 1996) expected for species lacking reliable asexual reproduction or aerial seed banks, the decline in recruit density in a fire/mast year to a distance of about 200 m from the fire edge should be strongly moderated by input from dead spruce within the burn area, and beyond 200 m should be nonzero, yet quite flat, as effectively all seeds, as with lodgepole pine, are by that distance derived locally from dead spruce. Second, at distances greater than 200 m from an unburned edge, we expect spruce seedling density to be positively correlated with local burnt ovulate cone density (as it would be with lodgepole pine).

## Methods

We used two recent fires within Kootenay National Park (50.8831°N, 116.0492°W) in the Rocky Mountains of eastern British Columbia, Canada. The park comprises mainly subalpine forest, at elevations ranging from 800 to 3400 m over an area of 1400 km^2^. The forest is dominated by lodgepole pine and Engelmann spruce, with small components of Douglas-fir (*Pseudotsuga menziesii* (Mirb.) Franco var. menziesii) and subalpine fir (*Abies lasiocarpa* (Hook.) Nutt.).

The Shanks fire occurred in 2001, when 2430 ha of forest burned on the southern slopes of Mount Shanks, beginning approximately 05 August 2001 and ending 04 September 2001. In 2003, large wildfires in the Tokumm Valley and on the slopes of Mount Verendrye joined to create a burned region of 17,409 ha, in what is now referred to as the Tokumm–Verendrye fire (Parks Canada 2004). The Tokumm–Verendrye fire burned between 31 July 2003 and 10 September 2003. Both fires were high intensity, stand-replacing fires, actively fought by Parks Canada and extinguished by rainfall. Both 2001 and 2003 were mast years for white spruce in the nearby Kananaskis Valley (Lobo and Millar 2013); the same trees were used in both years and produced on average 1.8 times more cones in 2003 than in 2001.

### Long transect: Shanks (2001) fire

We established a 637-m belt transect beginning at the edge of the unburned forest and proceeding into the burned area in a direction perpendicular to the fire edge. There were no residual (unburned) “islands” within 500 m of this transect. We counted Engelmann spruce seedlings along this transect in consecutive quadrats 0.5 by 2 m wide. To estimate local source strength at the time of the fire, we also tallied fallen ovulate cones within these quadrats, including those still attached to fallen branches. Because we conducted this study five years after the Shanks fire, almost no cones remained attached to standing burnt boles; we therefore did not estimate their density above the quadrats. At the center point of each 1 m^2^ quadrat, we recorded the charred organic layer depth.

### Short transects: Tokumm–Verendrye (2003) and Shanks (2001) fires

To quantify the abundance of cones (and thus of local seed supply) from burned trees and characterize the burned forest substrate, we placed 100-m belt transects at 43 sites within the Tokumm–Verendrye and Shanks burns, using the same procedure for counts of cones, seedlings, and organic layer thickness as in the Shanks long transect. We situated each short transect at least 300 m away from any living trees and ran them in a randomly chosen direction. Using maps of the daily area burned, we selected these 43 sites to represent as many different burn days as possible within the interval from ignition to extinguishment.

We compared observed seedling density of both spruce and pine at these short transects versus the product of cone density and the proportion of good (≤5 cm depth) seedbeds. For this (or any) small-seeded species, the cumulative survivorship on organic substrates >5 cm is essentially zero because the germinant cannot extend the radicle fast enough to maintain contact with the receding wetted perimeter during a short rainless period (Greene et al. 2007). That is, we expect a positive and significant relationship between recruit density and the product of cones and proportion of good seedbeds if the seed supply is completely local.

We examined the effect of fire date on regeneration by comparing date burned for short transects versus the scalar quantity: observed seedlings/m^2^ divided by the product of ovulate cones/m^2^ and proportion of seedbeds with an organic layer ≤5 cm. That is, we expect the denominator of the scalar to be proportional to the density of seedlings.

### Modeling the contributions of the living and burnt trees

The expected seedling density along the 630-m transect at Shanks has two components. First, there is the contribution from the living trees at the edge, which we express using the seed dispersal model of Greene and Johnson (1996) for the movement of wind-dispersed seeds into a clearing or burn. This model was originally calibrated using, among other species, Engelmann spruce in Kootenay National Park, as well as the dispersal data for this species from McCaughey and Schmidt (1987). This theoretical model for *N*(*x*), the seed density at distance *x* away from the perpendicular edge of an area source, depends on *Q*, the preabscission density of seeds (#/m^2^) produced within the area source, and an exponential term defining the dilution of the seed density with distance:


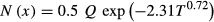
1

Equation (1) includes the nondimensional dispersal parameter, *T *=* *0.27*x*^0.814^*v*_*f*_ /(*az*_*h*_^0.954^), where *v*_*f*_ is the seed terminal velocity and *z*_*h*_ is the canopy height. These latter two values were 0.61 m s^−1^ and 29 m, respectively (Greene and Johnson 1996), for Engelmann spruce in this area. The wind parameters assume a forest canopy in full leaf; we ignored the fact that these burns had, at the time of dispersal, a large fraction of burnt trees moderately slowing the wind within the clearing. The empirical coefficients account for the increase in wind speed in the lee of the forest and with height above the ground (Greene and Johnson 1996). The nonexponential portion of the Greene and Johnson (1996) model is 0.5*Q*. Deep within the intact forest, the expected seed density on the ground would be *Q*; thus, 0.5*Q* represents the expected density at the forest edge.

One can extend Equation (1) to a model of seedling density (*N*_*seedlings*_) by assuming that seedbed-controlled survivorship is independent of distance. In a first approximation, assuming that all seeds perished within the fire and input could only come from the living trees at the edge, we set the nonexponential portion of Equation (1) as *F*, merely the observed mean of the first three seedling density values in the transect:
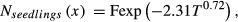
2

where the exponential term *T* is that from Equation (1). Equation (2) is a “straw man”; if our hypothesis is correct, we expect this model to greatly underpredict observed seedling density with increasing distance from the intact forest.

Now let us create a more complex model that includes the effect of aerial seeds surviving the passage of the flaming front. We followed Greene and Johnson (1998) in defining the cumulative juvenile survivorship (*S*) of a species with adequate light as:
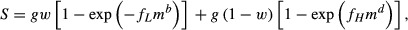
3

where *g* is survivorship through the granivory stage (0.43 per Greene and Johnson 1998), *w* is the fraction of good seedbeds (defined above as organic layer depth ≤5 cm), and *m* is the seed mass (0.0024 g for Engelmann spruce; D.F. Greene, unpublished data), which is allometrically related to germinant length. The empirical coefficients *f*_*L*_ = 1.83 and *f*_*H*_ = 0.33 are defined for good and poor (>5 cm depth) seedbeds, respectively, and the exponents *b *=* *0.43 and *d *=* *0.76 were determined empirically in Greene and Johnson (1998) for 25 gymnosperms and angiosperm tree species, including Engelmann spruce. Using the very high proportion of good seedbeds along the 630-m transect (*w *=* *0.899), we calculate *S* over the entire Shanks transect to be 0.049.

Given the mean cone density (*C*) from the transect and *R*, the mean number of viable seeds per cone (assumed to be 30 in a mass seeding year (Owens and Molder 1984)), we can recast the nonexponential portion of the area source equation as *CRS*. However, this estimate must be increased because the living trees produced a second mass crop in 2003 that was 1.8 times larger than in 2001. That is, our estimate of the seed source at the living edge is approximately 2.8 times higher than what it would have been immediately following the 2001 fire. Thus, for Shanks, the nonexponential term *F* in Equation (2) is replaced by 0.5 × 2.8*CRS*, or 1.4*CRS*. We assume that the non-mast years produce trivial amounts of seed and their input can therefore be ignored. Given the strongly skewed temporal distribution of crop sizes for this species (Greene and Johnson 2004), this is reasonable. For the contribution from the burnt trees in 2001, invariant with distance, we will add the expected seedling density given observed cone density and the proportion of good seedbeds. This amount must be reduced by the expected preabscission seed loss (*p*, as a surviving proportion) due to passage of the flaming front. The simulations of Michaletz et al. (2013) predicted a value of *p *=* *0.12. The final equation for seedling density, including contributions from both living and burnt trees, thus becomes:
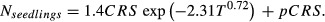
4

By inspection, the contribution from burnt trees at the edge (*x *=* *0) would be about 11.7 (1.4/*p*) times less than that from the living trees. In contrast, several hundred meters into the burn, the seedling density will have become essentially invariant (as the term exp(−2.31*T* ^0.72^) approaches 0), and the expected density is merely 0.12*CRS*. Note that the contribution from the burnt trees at the edge would in truth be only half of 0.12*CRS* because it cannot be matched by a burnt area source outside the fire perimeter. In any case, the input from the living trees will make superfluous any input from the burnt trees near the edge, and so, we will not bother to more seriously treat the decline in the burnt tree input as we move from deep in the fire interior to within a hundred meters of the edge.

Nonjuvenile lodgepole pines in this region have only serotinous cones, and thus, the expected pine seedling density is simply *pCRS*. According to de Groot et al. (2004), given typical flame durations, serotinous jack pine (essentially the same species as lodgepole) cones would have essentially *p *=* *1.0. The complement of viable pine seeds per cone prior to abscission, *R*, for this area is 21 (Greene, unpublished data). The number of cones per m^2^, *C*, will be provided empirically from the short transects. Finally, given a mean seed mass of 0.0033 g, *S* can be calculated for the short transects based on the proportion of good seedbeds (as with spruce).

### Seed viability window estimation

To determine whether the trees in the transects burned within the seed viability window, we calculated accumulated degree days (beginning ordinal day 1 = January 1) for each transect using the double-sine method with a lower threshold of 5°C (Zasada 1973; Allen 1976). We obtained maximum and minimum daily temperature data from 2001 and 2003 at the west gate of Kootenay National Park from Environment Canada (http://climate.weatheroffice.gc.ca). We then corrected temperature data for the elevation of each transect according to *T* = *T*_0_ − (4.2/1000)(*Z* − *Z*_0_) where *T* is the air temperature at elevation *Z* and *T*_0_ is the air temperature (°C) at elevation *Z*_0_ (m; 930 m for Kootenay west gate). The lapse rate used in this calculation is appropriate for elevation terrain change (where the atmospheric boundary layer is heated by the surface) and is thus lower than for air alone (Linacre and Geerts 1997).

## Results

**Figure 1:**
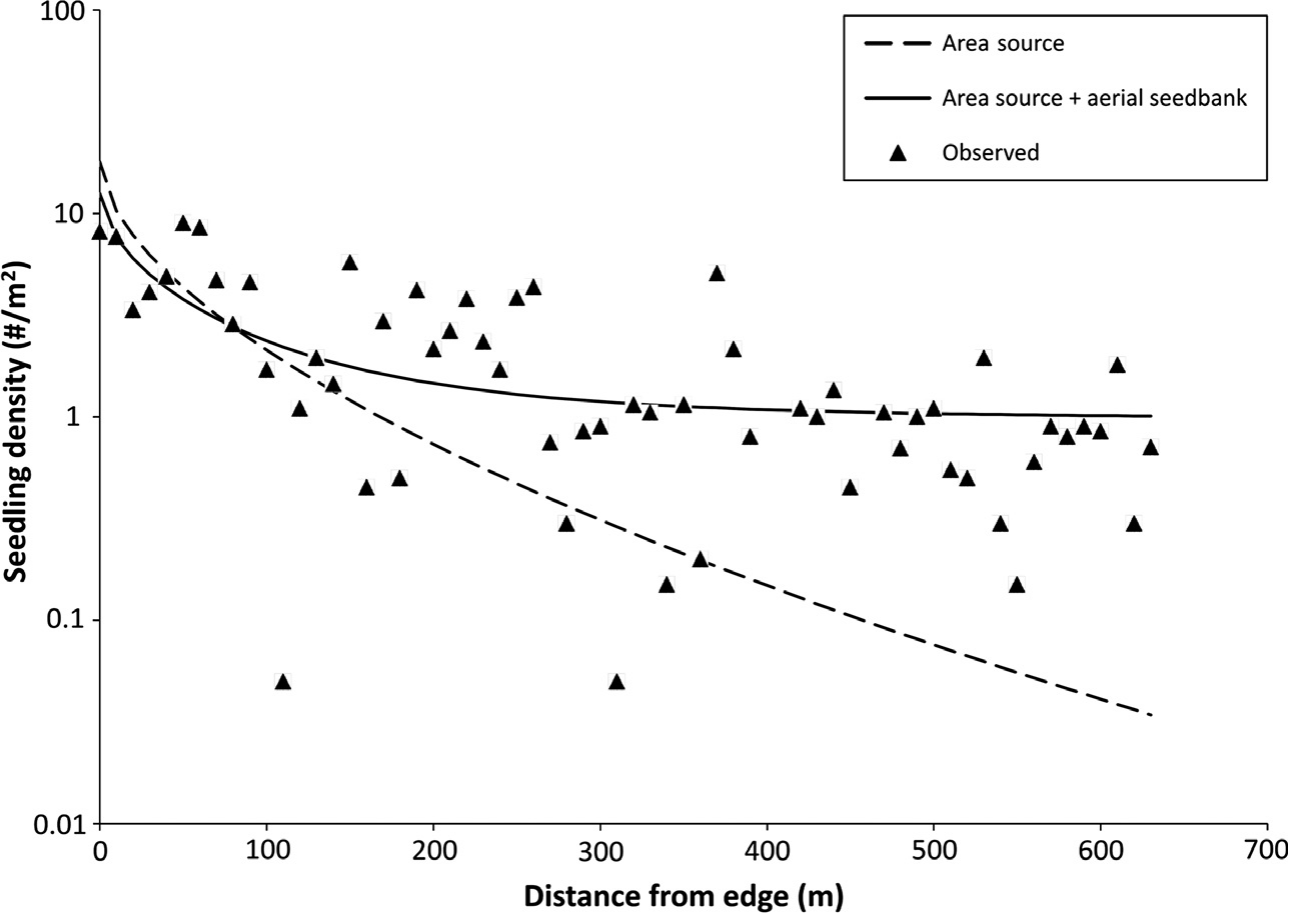
Observed spruce (*Picea glauca*) seedling density along the 637-m transect at Mount Shanks, compared to Equation (2) (the area source model of Greene and Johnson 1996) and to the modified area source model with a contribution from the burned trees (Equation (4)).

In the 637-m transect at the 2001 Shanks burn, seedling density decreased with distance from the burn edge as would be predicted by any model of seed dispersal by wind (Fig.1). There was no significant tendency for seedbeds to improve with distance; in fact, the proportion of good seedbeds decreased slightly with distance (*R*^2^ = 0.083, df = 62, *P* < 0.05), and thus, the unexpectedly modest decline in seedling density with distance must be due to an equally constrained decrease in seed input.

The area source model (no input from burnt trees) badly underpredicted the recruit density at large distances from the forest edge (Fig.1). However, the full model tended to overpredict densities. In short, neither model provided a normal distribution of residuals when predicted densities were regressed on observed densities. With a semi-log regression of predicted on observed values, we had a more reasonable residual distribution, finding that the full model better predicted observed recruit densities than the area source model (*R*^2^ = 0.549 or 0.475, respectively; df = 62; *P* < 0.0001 for either). Along this long transect, for *x* > 300 m, where the living trees were expected to have made a negligible contribution to recruitment, the overprediction of the full model was more acceptable: mean observed density of seedlings was 0.90 per m^2^, whereas we expected 1.07 from the burnt trees alone (= *pCRS* in the full model, Equation (4)).

**Figure 2:**
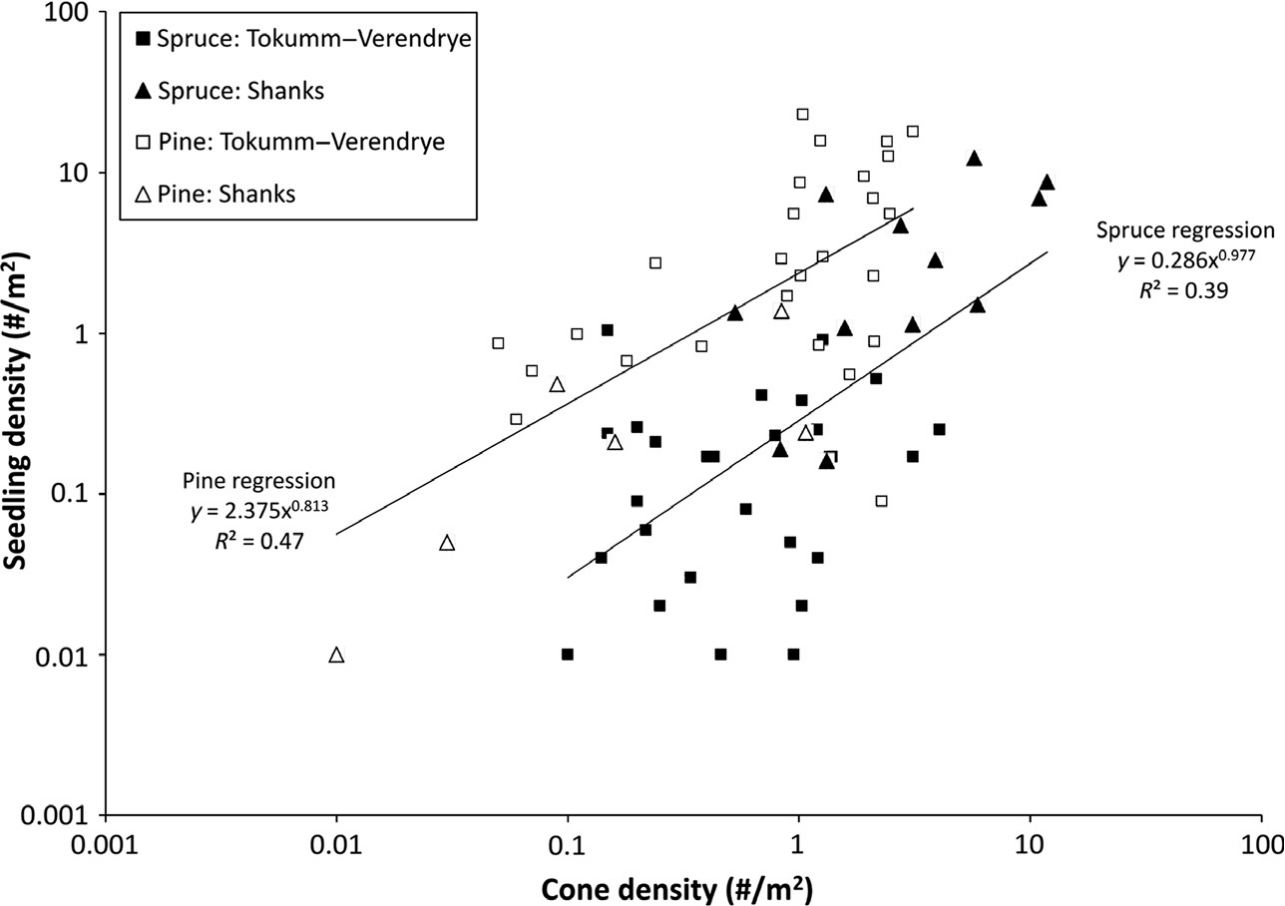
Relationship between spruce and pine (*Pinus contorta*) seedling density and cone density in the short (100 m) transects far from forest edges in the Shanks and Tokumm–Verendrye burns.

Several hundred meters from any edge, within both fires, the observed densities of spruce seedlings and cones on the short transects were well expressed by a log-log regression (*β* = 0.978, *P* < 0.001, df = 39, *R*^2^ = 0.391; Fig.2). In the subset of the same transects where pine was present, observed pine seedling density was also significantly related to cone density (*β *= 0.813, *P* < 0.001, df = 31, *R*^2^ = 0.471; Fig.2). There was no significant difference in the slopes for these two species, but the pine intercept was significantly higher than that of spruce (*t*-test; df = 68; *P* < 0.05), and the coefficient of determination (*R*^2^) was greater for pine.

**Figure 3:**
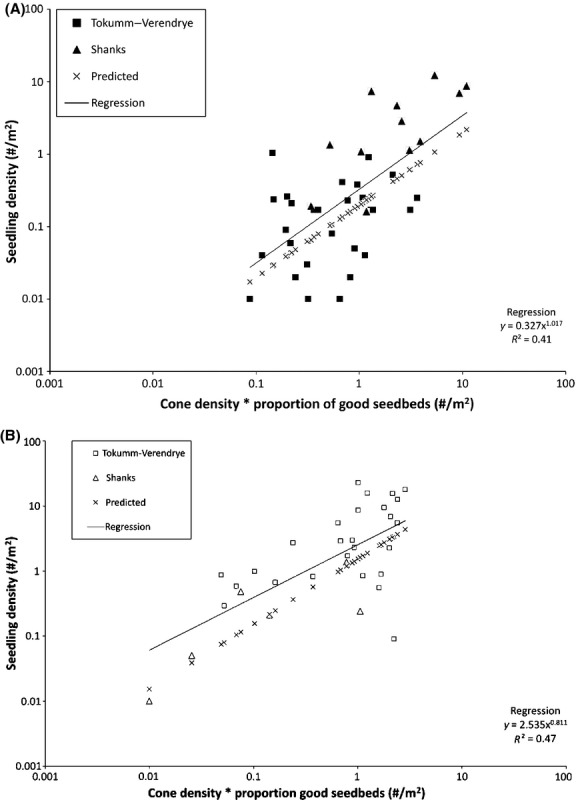
Seedling density of (A) spruce and (B) pine as a function of recruitment potential (the product of cone density and the proportion of good seedbeds) using the short (100 m) transects at the two fires, and our prediction (pCRS), based on the contribution by burned trees far from intact forest or residual stands (Equation (4)).

Does prediction of seedling density on the short transects improve when we use not just cone density but also seedbed quality? Predicting spruce seedling density using the product of cone density and the proportion of good seedbeds led to only a slightly higher positive correlation (*β *= 1.017, *P* < 0.001, df = 39, *R*^2^ = 0.414; Fig.3A); the effect of seedbed-mediated survivorship rendered unimportant because there was so little variation in the proportion of good seedbeds among transects. For spruce, regressing the seedling density predicted by our term for the contribution of the burned trees (*pCRS*) on the observed density for spruce gave a significant result (*R*^2^ = 0.491; df = 41; *P* < 0.0001) with a slope (1.00) not significantly different from 1, but an intercept (0.197) significantly larger than 0 (*t*-tests; *P* < 0.001); that is, the full model overpredicted recruitment density. Likewise, for pine, there was a significant relationship between observed seedling density and the product of the good seedbed proportion and local cone density (*β *= 0.8105, *P* < 0.001, df = 31, *R*^2^ = 0.469; Fig.3B) but a significant tendency to overpredict seedling density (*t*-tests on a regression of the *pCRS* prediction on observed seedling density). As with spruce, the slope of the prediction was 1.00; however, the intercept (1.515) was much higher than that for spruce (*P* < 0.001, df = 30, *R*^2^ = 1).

Adjusted for elevation, all of the areas in which we placed transects burned after the predicted mean degree day sum of 600 required for the onset of spruce seed germinability. However, 17 of the 43 short transects burned after the mean degree day date for the expected onset of cone opening (1177 degree days). Indeed, six of these transects burned as much as 10–15 days after the expected cone opening date. The 17 short transects burning after the presumed onset of cone opening did not have significantly lower ratios of predicted to observed seedling densities than the other transects (*t*-test; df = 38, *P* > 0.05).

**Figure 4:**
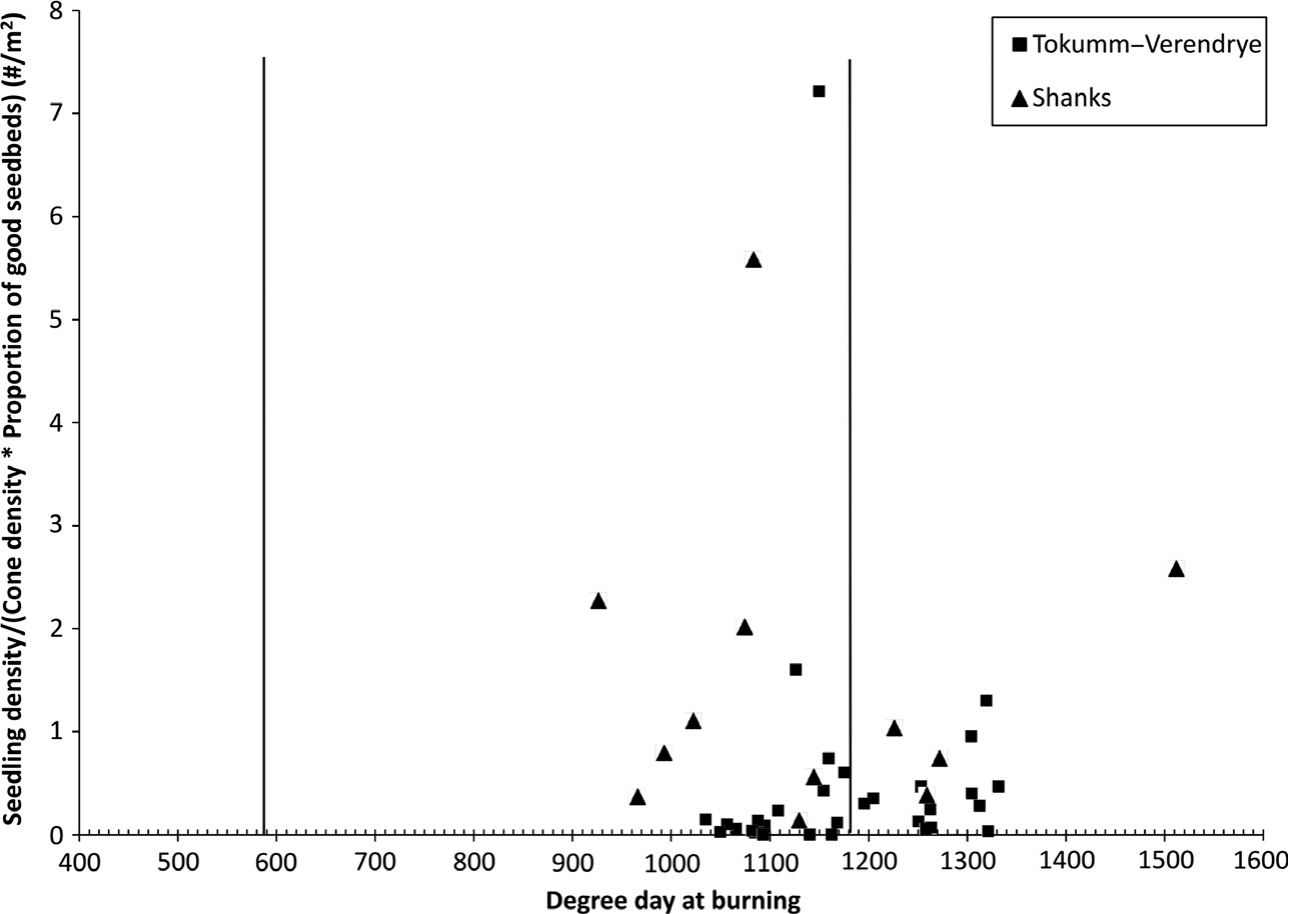
The effect of degree day on observed seedling density using a scalar (observed seedling density divided by the product of the proportion of good seedbeds and cone density) in the short transects. Vertical lines represent the boundaries of the viability window. As expected, none of our regeneration transects were on areas burned before the starting point (600 degrees). The end point (1180 degree days) is less well defined.

Statistically, fire dates had no effect on our scalar [seedling density/(cone density × proportion of good seedbeds)] in the short transects (Fig.4; *R*^2^ = 0.003, df = 41, *P* > 0.05). Likewise, elevation was unrelated to the scalar (*R*^2^ = 0.002).

## Discussion

Three kinds of evidence lead us to conclude that a large fraction of Engelmann spruce seeds survived the fires during these two mass seeding years. First, the flattening of the 0.6-km seedling dispersal curve is far more dramatic than has been seen in any dispersal curve for seeds or seedlings into a clear-cut or fire (c.f. the numerous studies cited in Greene and Johnson 1996). Further, we found no offsetting (and necessarily equally dramatic) amelioration of seedbeds with distance that could explain this seedling trend; indeed, as we saw, the quality of seedbeds instead modestly declined with distance. It seems likely therefore that a large fraction of the spruce seeds survived passage of the flaming fire front.

A second argument that a non-negligible fraction of spruce seeds survived the flames is that the short transects, located several hundred meters from any living sources, showed a strong positive relationship between cone density and seedling density; this relationship was only modestly improved when seedbed quality was included as a predictor of seedling density. There is no other explanation for this result except that in situ regeneration is occurring deep within the two fires.

Third, lodgepole pine, an aerial seed bank species, was similar to spruce in the response of its seedling density to cone density and seedbed quality. Indeed, the slopes for the two species were not significantly different. This similarity could occur only if seeds within the cones of both species survived the fire. That pine, as a serotinous species, is more strongly adapted to regeneration after fire is seen in its larger *R*^2^ values. The relation of seedling density to cone density and seedbed quality is slightly weaker in spruce, with regeneration depending on the fire coinciding with the viability window.

Engelmann and white spruce, important trees for forestry, have almost always been planted in clear-cuts because of the strong dispersal constraint (Greene et al. 2002a). But the density of spruce seedlings observed beyond 300 m at Shanks, around 1 m^−1^, is a value sufficient to be considered a fully stocked stand (i.e., with a minimum of 60% of 4-m^2^ quadrats having at least one spruce). These are astonishingly high recruitment densities for this species. Peters et al. (2005) found that with late spring fires (i.e., there can be no in situ regeneration) occurring during a mast year, white spruce regeneration within 100 m of an edge was far greater than with fires in non-mast years, but their reported recruit densities were far lower than what we have shown here (e.g., they found an average of 0.2 seedlings m^−2^ within the first 100 m from a burn edge for a 7-year-old fire; compare with Fig.1, a 6-year-old fire). To the best of our knowledge, the recruitment densities reported here are by far the greatest for white or Engelmann spruce in any burn at any distance from a fire edge.

For a given recruitment potential (i.e., the product of cone density and proportion of good seedbeds), pine had a seedling density that was approximately one order of magnitude higher than that of spruce (Fig.3). While pine has a larger seed and therefore better survivorship than spruce on any seedbed (e.g., Greene and Johnson 1998), we assume the main difference in seedling survivorship is due to seed survival through fire. Recall that in our model, we assumed that pine seeds would survive at about nine times the rate of spruce seeds (*P *=* *1.0 for pine, vs. *P *=* *0.12 for spruce). We assume this difference is primarily due to differences in the thermophysical properties of pine and spruce cones. We have unpublished data showing that seed embryo depth is about the same for the two species, suggesting that seed survival differences may then reflect differences in other thermophysical properties of cones such as mass density, water content, specific heat capacity, and/or thermal conductivity, but we yet lack the thermophysical data for pine cones that are available for white spruce cones.

The very simple argument *pCRS*, that is, that seedbed quality and deposited seed density should be the principal determinants of recruitment density, performed reasonably in determining the mean seedling densities in the short transects or in the last half of the Shanks transect. The slight tendency to overpredict recruitment far from the edge might be eliminated following subsequent field and laboratory work to improve our initial model parameter estimates. Particularly, the expected value of 0.12 seed survival during fire could be too high, or the cutoff of 5-cm organic layer depth defining good seedbeds could be made more stringent.

We had expected that cone opening would have a discernible effect on recruitment, with sites burning on the latest degree day dates having poorer transfire survival if the burn date occurred after the estimated cone opening date. At the onset of cone opening, the physical properties of cones change radically, providing far less heat transfer resistance and actually promoting cone combustion. Cone opening is driven by a gradual reduction in cone water content (Zasada 1978; Winston and Haddon 1981; Michaletz et al. 2013) combined with differential hygroscopic expansion of sclereid and fiber cells within cone scales (Dawson et al. 1997). As cone water content decreases and the scales reflex open, the surface-area-to-volume ratio of the cone increases while the thickness of exposed scales decreases. These open cones would then be classified as fine fuels and combust in a forest fire (Fonda and Varner 2004; Almeida et al. 2011), killing the seeds. Nonetheless, we found no deleterious effect of burning later. This makes us doubtful of the result of Michaletz et al. (2013); their suggested value of 1177 degree days for cone opening is based on subjective appraisals from a small number of studies, and it is seldom clear in those studies whether the recorded date is for all cones, the complete opening of only the first cone, the opening of the first few cone scales, etc. In short, while the lethal consequences for seeds of scale opening prior to a burn are undoubted, our results argue that the 1177 threshold is too low, and it is unlikely that this threshold can be refined until consistency in the definition of cone opening in abscission studies is attained.

If a fire occurs in a mast year, during the period when viable seed is still contained within closed cones, seeds surviving the fire would provide an in situ aerial seed bank for postfire recruitment. The capacity to occasionally behave as an aerial seed bank species would undoubtedly greatly increase the mean proportion of the landscape dominated by Engelmann or white spruce averaged over several centuries, but is this type of event so rare as to be unimportant? The proportion of total area burned during the period between 600 and 1177 accumulated degree days varies widely across the North American boreal forest, with estimates ranging from 0.14 at Schefferville, Quebec to 0.90 at Jasper, Alberta and a mean of 0.52 (Michaletz et al. 2013). Jasper is, like our two Kootenay NP sites, in the Rocky Mountains. Switching now to dates rather than degree days (as these would need to be calculated for each fire), we note that the last major fire in Kootenay NP prior to this was the August 1968 Vermilion Pass fire. Further south, the bulk of the area burned in the 1988 Yellowstone fires occurred in August. We suggest then that for the cordilleran forests of North America, late-summer fires are quite normal. Within the circumboreal forest, fire date generally occurs later in the growing season with increasing latitude. More generally in fire-prone systems, most of the area burned occurs toward the end of the dry season (Trapnell 1959; van Wilgen 1984; Wright and Agee 2004).

But of course, the availability of a *sizable* aerial seed bank requires a mast year and thus depends on the joint probability of fire during the period when germinable seed is contained in closed cones. In their study of the strongly right-skewed temporal distribution of annual seed crops for North American masting species, Greene and Johnson (2004) defined a mast year such that the probability was about 0.1. Thus, quite speculatively, if in the North American boreal forest 50% of the area burned is in the late summer, then the probability of this aerial seed bank behavior occurring in a given fire would be 0.05 (=0.5 × 0.1). In conclusion, this capacity to reproduce in situ from dead trees will not be common but it will not be negligible either.

One notes that the semi-serotinous black spruce (*Picea mariana*) in the North American boreal forest has seeds that routinely survive fire (with a survival rate around 0.5: Greene et al. 1999), even though the physical properties and radial heat transfer characteristics of its cones are similar to those of Engelmann spruce cones (Parker and McLachlan 1978). On a mature black spruce, the persistent cones are held in a large mass near the top of the stem. Two factors might account for the higher seed survival rates in black spruce versus Engelmann spruce. First, black spruce cones are located at the top of the canopy and, as described by classical plume theory (Mercer and Weber 2001), are consequently exposed to lower plume temperatures than Englemann spruce cones, which span a relatively larger range of canopy heights (Greene et al. 2002b). Second, in forest fires, this clustering probably reduces the heat flux incident on cone surfaces and thus provides even greater resistance to heat transfer than is provided by the individual cones themselves. During a mast year, Engelmann and white spruce cones are also held in dense clusters along branches in the upper third of the crown; thus, it is expected that this clustering likewise enhances heat transfer resistance in these species. That is, in a mast year, the survival of spruce seeds may be higher than simulations using individual cones suggest.

In the small number of cases where it has been examined, it has been shown that clustering promotes seed survival through fire in non-serotinous species. In Australia, small *Kunzea* (Myrtaceae) capsules experimentally heated by Judd and Ashton (1991) survived extreme heat longer within clusters than when isolated, and Whelan and Brown (1998) found that clustering in *Callistemon citrinus* (Myrtaceae) increased the proportion of viable seed at the lower end of expected wildfire temperatures. This suggests that perhaps even small, nonwoody fruits produced in large clusters in a mast year by conifers such as *Callitris* (Takaso and Tomlinson 1989), *Juniperus* (Jordano 1993), *Dacrydium* (Kelly 1994) may have a non-negligible fraction of seeds surviving a fire, particularly if it is of low intensity. Parenthetically, clustering can also aid seed survival in serotinous species. Bradstock et al. (1994) found differential seed survival between species and populations in experimentally heated *Hakea* (common in fire-prone regions of Australia and South Africa) and concluded that the penetration of lethal heat was influenced by the position and degree of exposure of the pods.

In light of our results, it is worth reconsidering the evolution of masting in fire-prone regions. The two dominant hypotheses about the selective value of masting, both related to economies of scale (Kelly 1994), relate to predation and pollination by wind. The first is that the predation rate is inversely related to the crop size because the granivorous populations cannot track the essentially random occurrences of mast years (Silvertown 1980). The second argument notes that masting, in the higher latitudes at least, primarily occurs among anemophilous species (Herrera et al. 1998) and is true of not just female function but male as well. Given the presumably much less efficient transfer of pollen by wind than animals, masting has been selected for to ensure full seed set. We advance a third hypothesis: that in fire-prone regions, masting will occasionally permit a fraction of the seeds to survive, and thus, the dispersal constraint imposed by very large, stand-replacing fires will be mitigated as with an aerial seed bank strategy. Masting is crucial for these species as (1) the expected surviving fraction of the seed crop during fire will be low because the investing structures will not yet be as dry as with typical serotinous species and (2) masting permits dense clustering of fruits or cones which in turn should increase the mean seed survival rate. Like the pollination efficiency argument, this hypothesis is limited in scope, in this case to regions where fire is the prevailing disturbance. It nonetheless presents one more mechanism by which economy of scale acts as a selective force for masting.
